# The Effects of Glycogen Synthase Kinase-3beta in Serotonin Neurons

**DOI:** 10.1371/journal.pone.0043262

**Published:** 2012-08-17

**Authors:** Wenjun Zhou, Ligong Chen, Jodi Paul, Sufen Yang, Fuzeng Li, Karen Sampson, Jim R. Woodgett, Jean Martin Beaulieu, Karen L. Gamble, Xiaohua Li

**Affiliations:** 1 Department of Psychiatry and Behavioral Neurobiology, University of Alabama at Birmingham, Birmingham, Alabama, United States of America; 2 Department of Physic, Faculty of Science and Engineering, Laval University, Québec City, Québec, Canada; 3 Samuel Lunenfeld Research Institute, Toronto, Ontario, Canada; 4 Department of Psychiatry and Neuroscience, Faculty of Medicine, Laval University, Québec City, Québec, Canada; University of Iowa, United States of America

## Abstract

Glycogen synthase kinase-3 (GSK3) is a constitutively active protein kinase in brain. Increasing evidence has shown that GSK3 acts as a modulator in the serotonin neurotransmission system, including direct interaction with serotonin 1B (5-HT1B) receptors in a highly selective manner and prominent modulating effect on 5-HT1B receptor activity. In this study, we utilized the serotonin neuron-selective GSK3β knockout (snGSK3β-KO) mice to test if GSK3β in serotonin neurons selectively modulates 5-HT1B autoreceptor activity and function. The snGSK3β-KO mice were generated by crossbreeding GSK3β-floxed mice and ePet1-Cre mice. These mice had normal growth and physiological characteristics, similar numbers of tryptophan hydroxylase-2 (TpH2)-expressing serotonin neurons, and the same brain serotonin content as in littermate wild type mice. However, the expression of GSK3β in snGSK3β-KO mice was diminished in TpH2-expressing serotonin neurons. Compared to littermate wild type mice, snGSK3β-KO mice had a reduced response to the 5-HT1B receptor agonist anpirtoline in the regulation of serotonergic neuron firing, cAMP production, and serotonin release, whereas these animals displayed a normal response to the 5-HT1A receptor agonist 8-OH-DPAT. The effect of anpirtoline on the horizontal, center, and vertical activities in the open field test was differentially affected by GSK3β depletion in serotonin neurons, wherein vertical activity, but not horizontal activity, was significantly altered in snGSK3β-KO mice. In addition, there was an enhanced anti-immobility response to anpirtoline in the tail suspension test in snGSK3β-KO mice. Therefore, results of this study demonstrated a serotonin neuron-targeting function of GSK3β by regulating 5-HT1B autoreceptors, which impacts serotonergic neuron firing, serotonin release, and serotonin-regulated behaviors.

## Introduction

Glycogen synthase kinase-3 (GSK3) [1] is widely distributed in brain [2] and is an influential enzyme in many aspects of neuronal function [3], [4]. Unlike other protein kinases, GSK3 is highly active under resting conditions, and undergoes inhibitory regulation upon cellular stimulation, such as by neurotrophins and several neurotransmitters [5], [6], [7]. Cumulative evidence from *in vitro* measurements, pharmacological studies, and animal behavioral tests strongly supports GSK3 as an integrative mediator of serotonin neurotransmission [8]. GSK3 is also a prominent pharmacological target of major psychotropic drugs, including lithium [9], [10], serotonin-modulating antidepressants [5], [11], and antipsychotics [12], [13], [14], all of which act directly or indirectly to suppress GSK3 activity.

As a protein kinase, GSK3 phosphorylates many protein substrates that distribute in both neural and peripheral tissues [3], [15]. The two isoforms of GSK3, GSK3α and GSK3β [16], are subject to similar regulation but may have distinguishable functions [17], [18], [19], [20]. As a result, *in vivo* activation or inhibition of GSK3α and GSK3β can have a variety of physiological effects. Thus, identifying GSK3 substrates that have specific functions in brain circuits could be critical in understanding the neurological actions of GSK3 and developing GSK3-targeting therapeutics.

We have identified GSK3β, but not GSK3α, as a direct modulator of 5-HT1B receptors [21], [22]. Both human and mouse 5-HT1B receptors contain eight GSK3 consensus phosphorylation sites [23] that locate in intracellular loops 1, 2, and 3 of the G protein-coupled receptor (GPCR) [8], [22]. In contrast, the highly homologous 5-HT1A receptors have no GSK3 consensus sites in intracellular loops 1 and 2, and the two sites in intracellular loop-3 of human 5-HT1A receptors are not homologous with mouse 5-HT1A receptors. In cultured heterologous cells expressing 5-HT1B or 5-HT1A receptors, GSK3β directly associates with 5-HT1B receptors at the [pS(154)AKRpT(158)] sequence located in the i2-loop [22]. GSK3β acts as a signal pathway-selective modulator of 5-HT1B receptors [21]. Disrupting the interaction between 5-HT1B receptors and GSK3β by mutagenesis or GSK3 inhibitors effectively abolishes the coupling of Giα2 to 5-HT1B receptors [21] and serotonin-induced inhibition of cAMP [22], but not receptor recruitment of β-arrestin2 [22]. The effect of GSK3β on 5-HT1B receptors is to facilitate receptor activity, which is opposite to other GPCR-regulating protein kinases (GRKs) that typically desensitize and terminate GPCR activity [24]. This is a highly selective function of GSK3β, as it does not alter regulation of Giα2 and cAMP by 5-HT1A receptors [21], [22].

In mouse brain tissues, pre-treatment of cortical slices with GSK3 inhibitors completely abolishes the inhibitory effect of 5-HT1B receptors on potassium-evoked ^3^H-serotonin release [21], a characteristic function of 5-HT1B autoreceptors in the axon terminals of serotonin neurons [25], [26], [27]. GSK3 inhibitors also facilitate the anti-immobility effect of 5-HT1B receptor agonist in the tail suspension test [21], but has little effect in 5-HT1B receptor agonist-induced increase in horizontal activity in the open field, suggesting that GSK3 has differential effects in modulating 5-HT1B receptor-regulated behaviors.

5-HT1B receptors share high protein sequence homology and signal transduction processes with 5-HT1A receptors, and both act as autoreceptors and heteroreceptors in the brain [28], [29], [30], [31]. Different from 5-HT1A autoreceptors that are located in the somatodendrites to reduce neuron firing and serotonin output [27], 5-HT1B autoreceptors are primarily located on the serotonin neuron axon terminals that extend to other brain regions. Upon activation, 5-HT1B autoreceptors cause a strong feedback inhibition of serotonin release [27]. Meanwhile, 5-HT1B receptors also act as heteroreceptors in several brain regions to control release of other neurotransmitters [26], rendering complex physiological and behavioral effects. Therefore, to further understand the functional impact of GSK3β, that is also widely distributed in brain, in modulating 5-HT1B receptors, it is important to dissect the function of GSK3β in regulating 5-HT1B autoreceptors separately from its heteroreceptors. In this study, we utilized serotonin neuron-selective GSK3β knockout (snGSK3β-KO) mice to examine the effect of GSK3β on 5-HT1B autoreceptors.

## Results

### 1. Serotonin neuron-selective GSK3β knockout (snGSK3β-KO) mice

**Figure 1:**
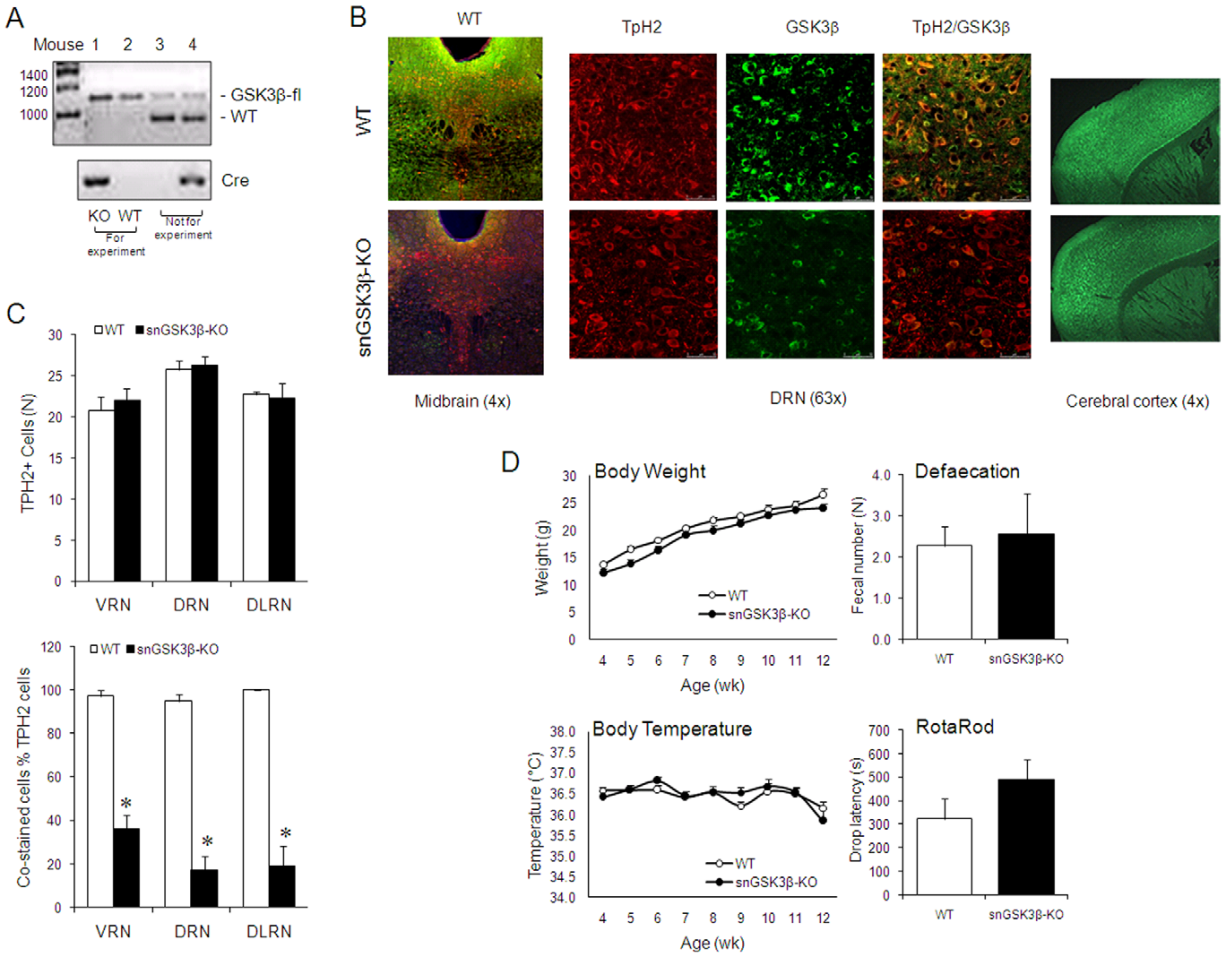
Characterization of snGSK3β-KO mice. (A) Tail DNA genotyping of GSK3β-flox and Cre for selecting snGSK3β-KO and littermate WT mice. Lane 1, GSK3β-flox homozygote and ePet1-Cre (+)  =  GSK3β knockout (KO); Lane 2, GSK3β-flox homozygote and ePet1-Cre (-)  =  GSK3β wildtype (WT); Lane 3, GSK3β-flox heterozygote and ePet1-Cre (-); Lane 4, GSK3β-flox heterozygote and ePet1-Cre (+). Mice with genotypes in Lane 1 and Lane 2 were used for experiments. (B) Immunostain of TpH2-positive cells (red), GSK3β-containing cells (green), and GSK3β/TpH2 co-stained cells in the midbrain (4x magnification) and the dorsal raphe (DRN) (63x magnification), and immunostain of GSK3β-containing cells (green) in the cerebral cortex (4x magnification). (C) quantification of immunostained TpH2-positive cells (upper panel) and GSK3β/TpH2 co-stained cells (lower panel) in the ventral raphe (VRN), dorsal raphe (DRN), and dorsal lateral raphe (DLRN) areas. Upper panel: TpH2-positive neurons were counted to represent the number of serotonin neurons in each area. No statistically significant difference (*p*>0.05, n = 4–5 per genotype) in Student's t-test when each area of the two genotypes was compared. Lower panel: GSK3β in GSK3β/TpH2 co-immunostained cells were calculated as percent of total TpH2-positive cells in each area. *p<0.05, n = 4–5 per genotype, in Student's t-test when each area of the two genotypes was compared. (D) Phenotypic comparisons of body weight, body temperature, defecation, and Rotarod movement between WT and snGSK3β-KO mice. Mean ± SEM; Mixed model analysis showed no significant difference between the two genotypes in body weight or body temperature (*p*>0.05, n = 26–30 per genotype); Student's t-test showed no significant difference between the two genotypes in defecation (*p*>0.05, n = 7 per genotype) and Rotarod movement (*p*>0.05, n = 5 per genotype).

To study the function of GSK3β in serotonin neurons, GSK3β was selectively deleted from serotonin neurons (snGSK3β-KO mice) by crossbreeding GSK3β-flox/flox mice [32] with ePet1-Cre mice [33]. Homozygous male snGSK3β-KO mice and littermate WT mice were selected by tail DNA genotyping of offsprings of GSK3β-flox/flox and GSK3β-flox/flox:ePet1-Cre mating pairs (Figure 1A).

TpH2-containing serotonin neurons were visible in the midbrain raphe nucleus in both WT and snGSK3β-KO mice (Figure 1B). Quantification of TpH2-positive neurons in the ventral, dorsal, and dorsal lateral raphe nucleus of the midbrain showed that the numbers of serotonin neurons were almost the same in WT and in snGSK3β-KO mice (Student's t-test; t(7)_VRN_ = −0.497, t(6)_DRN_ = −0.237, t(6)_DLRN_ = 0.221; *p*>0.05 for all; Figure 1B, 1C). In contrast, GSK3β immunostaining in the same raphe areas was stronger in WT mice than in snGSK3β-KO mice (Figure 1B, 1C). Quantification of GSK3β and TpH2 co-immunostained neurons as the percentage of total TpH2-positive neurons in the ventral, dorsal, and dorsal lateral raphe nucleus of snGSK3β-KO mice showed a significant reduction to approximately 65%, 85%, and 80%, respectively, when compared to WT mice (Student's t-test; t(7)_VRN_ = 9.39, t(6)_DRN_ = 10.63, t(3)_DLRN_ = 8.90; *p*<0.05 for all; Figure 1B, 1C). It was noted that some GSK3β-positive cells remained in the raphe area (Figure 1B), which may represent either non-complete GSK3β knockout in some TpH2-positive neurons, GSK3β in Pet1 non-expressing cells, or GSK3β in non-serotonergic cells. GSK3β immunostaining in the cerebral cortex did not identify a remarkable difference between WT and snGSK3β-KO mice (Figure 1B).

Unlike the global knockout of GSK3β that is lethal to animals [34], all snGSK3β-KO mice lived into adulthood when they were used for experiments (8–14 weeks old). Continued observation also showed that both male and female snGSK3β-KO breeders survived beyond 9 months of age. Body weight and body temperature recorded during 4–12 weeks of age showed no significant differences between snGSK3β-KO and WT mice (mixed models; the main effects and interaction for body weight are F (1,170)_genotype_ = 2.59, *p*_genotype_ = 0.1092; F(8,170)_age_ = 150.53, *p*_age_<0.0001, F(8,170)_interaction_ = 1.07, *p*_interaction_ = 0.3890; the main effects and interaction for body temperature are F(1,173)_genotype_ = 0.31, *p*_genotype_ = 0.5797, F(8,173)_age_ = 2.61, *p*_age_ = 0.01, F(8,173)_interaction_ = 1.05, *p*_interaction_ = 0.4 respectively; Figure 1D). Defecation recorded at 12 weeks of age also had no significant difference between snGSK3β-KO and WT mice (Student's t-test; t(30)_def_ = 0.29, *p* = 0.7758; Figure 1D). snGSK3β-KO mice performed normally as WT littermates in the Rotarod test (Student's t-test; t(6)_Rotarod_ = −1.47, *p* = 0.1926; Figure 1D). Therefore, snGSK3β-KO mice appeared relatively normal compared to their littermate WT controls.

**Figure 2:**
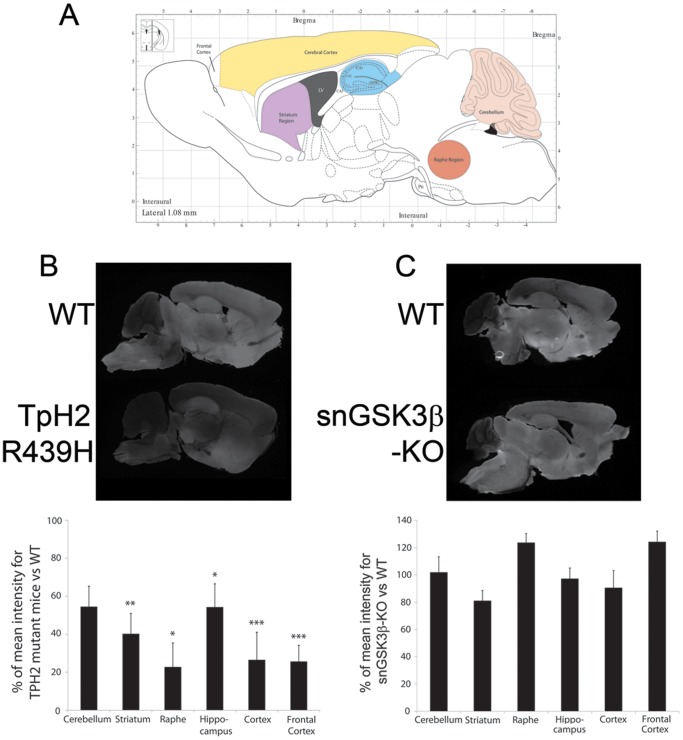
Measurement of serotonin tissue content variations in different brain areas using quantitative near infrared fluorescence imaging of serotonin immunolabeling. (A) Schematic representation of the regions that were selected for analysis [67]. (B) Imaging of serotonin immunofluorescence signal and quantification of relative changes in serotonin content in sagital brain section of TpH2 R439H mice along with sections from the corresponding littermate WT control analyzed within the same scan. (C) Imaging of serotonin immunofluorescence signal and quantification of relative changes in serotonin content in snGSK3β-KO mice along with sections from its corresponding littermate WT control analyzed within the same scan. Average signal value for WT control animals was normalized to 100%, data are mean ± SEM from six sections per genotype. *p*≤0.05*, 0.01** or 0.005*** in Student's t-test when each mutant genotype was compared to its corresponding littermate WT mice.

To ensure that deletion of GSK3β expression in raphe neurons did not lead to serotonin depletion in the brain, we used quantitative near-infrared immunofluorescence imaging [35] to measure relative fold variations in serotonin levels between mice of different genotypes. This approach allowed us to quantify reductions of 60–80% in serotonin levels in all tested brain areas (Figure 2A) of mice expressing a R439H loss-of-function mutant form of TpH2 (Figure 2B), which is comparable to the fold-change obtained by HPLC measurement in these mice [11]. In contrast, no reduction of serotonin levels was observed between WT and snGSK3β-KO mice (Figure 2C), and therefore suggesting that inactivation of GSK3β does not cause overt deficiencies of serotonin neurons and their capacity to synthesize serotonin.

### 2. Dorsal raphe serotonin neuron firing in snGSK3β-KO mice

**Figure 3:**
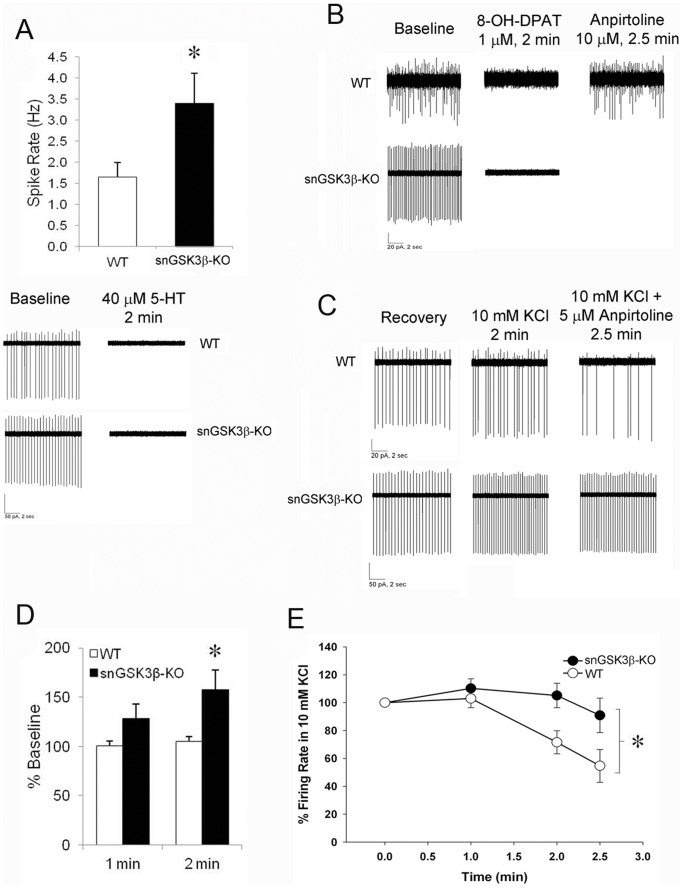
Dorsal raphe serotonin neuron firing and response to 5-HT1B and 5-HT1A receptor agonists. (A) Baseline spontaneous firing in ventral medial dorsal raphe serotoninin neurons from WT and snGSK3β-KO mice. Mean ± SEM, N = 9–10 neurons/genotype. *p<0.05, Student's t-test; Lower panel is representative traces of firing rate from WT (top) and snGSK3β-KO (bottom) serotonin neurons at baseline and during 2 min serotonin (5-HT, 40 µM) bath application. (B) Representative traces of firing rate from WT (top) and snGSK3β-KO (bottom) serotonin neurons at baseline (left) and after bath application of 1 µM 8-OH-DPAT for 2 min (middle) and 10 µM anpirtoline for 2.5 min. (C) Representative traces of firing rate from WT (top) and snGSK3β-KO (bottom) serotonin neurons, from left to right: post-serotonin recovery, 10 mM KCl (2 min), and 5 µM anpirtoline (2.5 min) in the presence of 10 mM KCl. (D) Change in neuronal firing after 1 and 2 min application of 10 mM KCl in WT and snGSK3β-KO neurons, expressed as percent of baseline firing rate. Mean ± SEM, two-way ANOVA *p*<0.05 for the main effects of Genotype, Time, and the interaction, *p<0.05 compared to WT, Sidak post hoc test. (E) Time-dependent response to anpirtoline (5 µM) in WT and snGSK3β-KO neurons, expressed as the percentage of spike rate after 2 min of 10 mM KCl. N = 9–10 neurons, mean ± SEM, two-way ANOVA, *p<0.05 for Genotype main effect. In addition, there was a main effect of Time such that all three time points were significantly different from one another (*p*<0.05, Sidak post hoc test).

To test the effect of GSK3β on serotonergic neuronal activity, we used loose-patch electrophysiological recordings of serotonin neurons in the dorsal raphe nucleus (DRN) of WT and snGSK3β-KO mice. Serotonin neurons were selected for recording based on a 50% or greater reduction in spontaneous action potential frequency following a 2 min treatment of serotonin (40 µM) [36]. The mean spontaneous action potential frequency of serotonin neurons from snGSK3β-KO mice at baseline was twice the mean baseline firing rate of neurons from WT mice (Student's t-test; t(17) = 2.45, *p*<0.05; Figure 3A). Serotonin application silenced neuronal firing equally in both genotypes (Figure 3A). Furthermore, both WT and snGSK3β-KO neurons were completely silenced by the 5-HT1A receptor agonist, 8-OH-DPAT (1 µM) (Figure 3B). Unlike serotonin and 8-OH-DPAT, 2 min application of the 5-HT1B receptor agonist anpirtoline had no effect on the spontaneous firing of WT serotonin neurons in the DRN (Figure 3B).

In order to test the effect of GSK3β on 5-HT1B autoreceptor activity in the DRN serotonin neurons, we next used a high concentration of potassium chloride (KCl, 10 mM) to depolarize neurons [37] prior to application of anpirtoline. A two-way repeated measures ANOVA (Genotype X Time) of the firing rate following 1- and 2-min application of potassium revealed significant main effects of genotype, time, and a significant interaction (F(1,17) = 5.71, F(1,17) = 17.26, and F(1,17) = 9.49, respectively; *p*<0.05 for all). Specifically, the higher concentration of potassium increased neuronal firing of snGSK3β-KO neurons by 29% and 58% above baseline after 1 and 2 min of exposure, respectively, but did not significantly increase the firing rate above baseline activity in WT neurons (Sidak post hoc, *p*<0.05; Figure 3C, 2D). Unlike spontaneous neuronal firing, however, high potassium-induced neuron firing was significantly suppressed upon activation of 5-HT1B receptors by anpirtoline in WT neurons, whereas the response to anpirtoline was significantly diminished in snGSK3β-KO neurons (two-way ANOVA; main effects of Genotype and Time, F(1,17) = 5.42, *p*<0.05 and F(1,17) = 14.14, *p*<0.05, respectively, but the interaction failed to reach significance, F(1,17) = 3.16, *p* = 0.092; Figure 3C-3E).

### 3. Regulation of forskolin-stimulated cAMP by 5-HT1 receptors in the cerebral cortex of snGSK3β-KO mice

**Figure 4:**
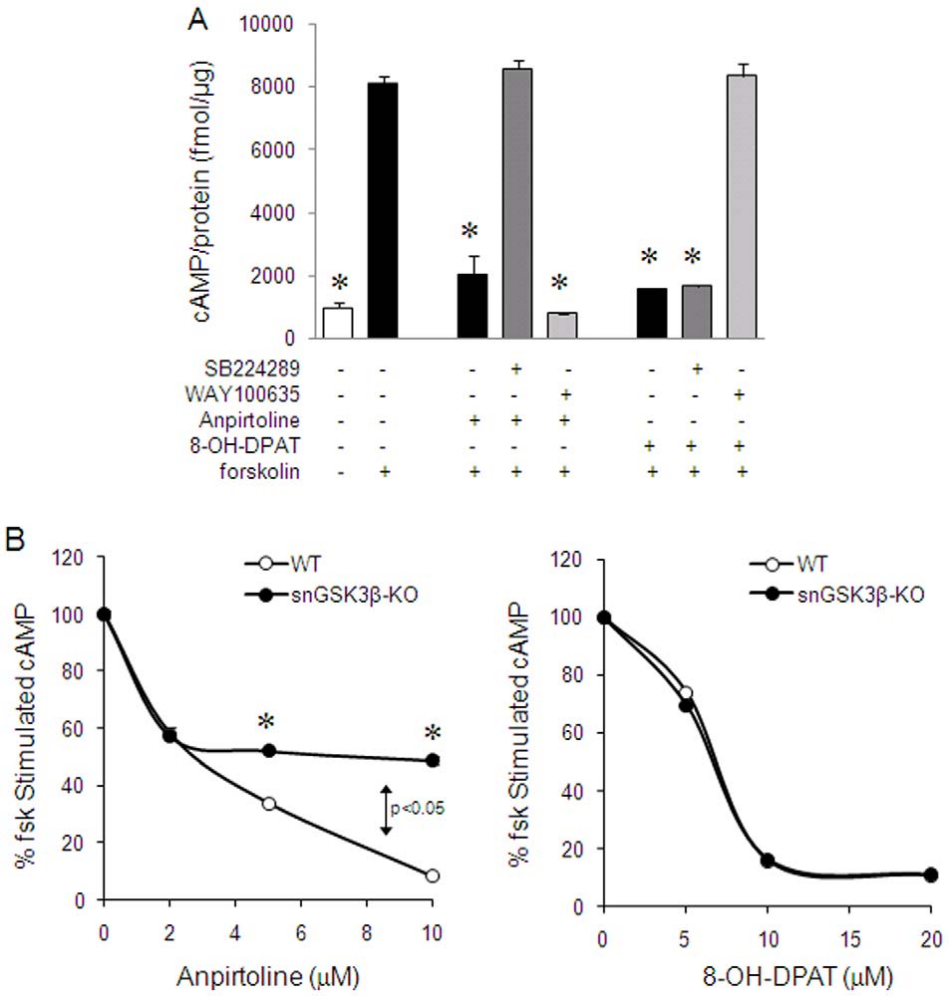
Regulation of forskolin-stimulated cAMP by 5-HT1B and 5-HT1A receptor agonists in the cerebral cortical slices. (A) Cerebral cortical slices from WT mice were pre-treated without or with the 5-HT1B receptor antagonist SB224289 (5 µM) or the 5-HT1A receptor antagonist WAY100635 (5 µM) for 15 min, followed by anpirtoline (10 µM) or 8-OH-DPAT ((10 µM) for 30 min, and then treated with forskolin (10 µM) for 15 min. Mean ± SEM, n = 3 individual experiments. *p<0.05 in LSD post hoc of one way ANOVA when values were compared to forkolin treatment alone. (B) Cortical slices from WT and snGSK3β-KO mice were treated with anpirtoline or 8-OH-DPAT for 30 min at indicated concentrations followed by an additional 15 min treatment with forskolin (10 µM). Mean ± SEM, n = 4. *p<0.05 in LSD post hoc of two way ANOVA when values of each anpirtoline concentration in snGSK3β-KO slices were compared to that in WT slices. *p*>0.05 in LSD post hoc of two way ANOVA when values of each 8-OH-DPAT concentration in snGSK3β-KO slices were compared to that in WT slices.

Both 5-HT1B and 5-HT1A receptors couple to Gi protein-linked inhibition of cAMP [38], and we have previously shown that inhibition of GSK3β in cells and brain slices abolishes 5-HT1B receptor-, but not 5-HT1A receptor-induced inhibition of cAMP production [21], [22]. To test if selective deletion of GSK3β from serotonin neurons affects cAMP production, we measured forskolin-stimulated cAMP production in the cerebral cortex that is innervated by serotonergic axons. In the WT cortical slices, both the 5-HT1B receptor agonist anpirtoline and the 5-HT1A receptor agonist 8-OH-DPAT reduced forskolin-stimulated cAMP production (one way ANOVA; F(7,18) = 104.32, *p*<0.001; LSD post hoc; Figure 4A). The effect of anpirtoline was blocked by the 5-HT1B receptor antagonist SB224289 (LSD post hoc, *p*<0.05; Figure 4A), but not by the 5-HT1A receptor antagonist WAY100635 (LSD post hoc, *p*>0.05; Figure 4A), whereas the effect of 8-OH-DPAT was blocked by WAY100635 (LSD post hoc, *p*<0.05; Figure 4A) but not by SB224289 (LSD post hoc, *p*>0.05; Figure 4A), confirming the selective effects of anpirtoline and 8-OH-DPAT on 5-HT1B receptors and 5-HT1A receptors, respectively. In WT mice, the effect of anpirtoline on forskolin-stimulated cAMP was concentration-dependent, with 2 µM causing 50% reduction, and 10 µM achieving near maximal inhibition (two-way ANOVA; F(3,25)_treatment_ = 1415.91, F(1,25)_genotype_ = 307.78, and F(3,25)_interaction_ = 141.08, *p*<0.0001 for all effects; LSD post hoc, *p*<0.05; Figure 4B, left panel). In snGSK3β-KO mice, a low concentration of anpirtoline (2 µM) was able to reduce 50% of cAMP production (two-way ANOVA; LSD post hoc, *p*<0.05; Figure 4B, left panel), similar to the WT mice. However, the effect of anpirtoline plateaued at 2 µM, and higher concentrations of this compound were unable to elicit additional inhibition (LSD post hoc; *p*>0.05; Figure 4B, left panel). In contrast to the limited effect of anpirtoline in snGSK3β-KO mice, 8-OH-DPAT effectively caused concentration-dependent inhibition of forskolin-stimulated cAMP production in both WT and snGSK3β-KO mice, and this effect was the same in both genotypes (two-way ANOVA; F(3,25)_treatment_ = 6182.19, *p*<0.0001; F(1,25)_genotype_ = 2.35, *p* = 0.1386, F(3,25)_interaction_ = 4.55, *p* = 0.0116; Figure 4B, right panel).

### 4. Regulation of serotonin release by 5-HT1 receptors in the cerebral cortex of snGSK3β-KO mice

**Figure 5:**
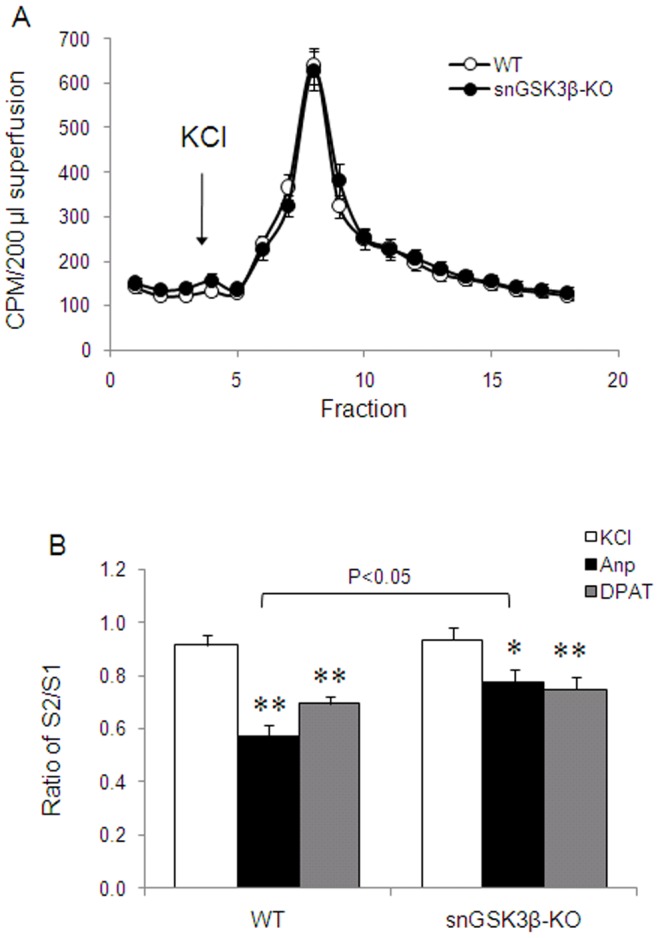
Regulation of serotonin release by 5-HT1B and 5-HT1A receptor agonists in the cerebral cortical slices. (A) Potassium chloride (KCl, 50 mM)-evoked [^3^H]-serotonin release was measured in freshly isolated cerebral cortical slices of WT and snGSK3β-KO mice. Mean ± SEM, n = 22 individual treatment. (B) Serotonin release was stimulated by potassium chloride (KCl, 50 mM) twice. Prior to the second KCl stimulation, slices were treated with anpirtoline (Anp, 5 µM) or 8-OH-DPAT (DPAT, 1 µM) for 5 min. Data is expressed as percent of baseline [^3^H]-serotonin release (fractions 1–3). Mean ± SEM, n = 6–13 in each treatment. *p<0.05, **p<0.01 in LSD post hoc of two way ANOVA when treatment was compared to baseline in the same genotypes. The response to anpirtoline in WT and snGSK3β-KO mice was significantly different (*p*<0.05).

A characteristic function of 5HT1 autoreceptors is to negatively regulate serotonin release [26], [27], and GSK3 inhibitors were able to reverse the inhibitory effect of the 5-HT1B receptor agonist anpirtoline on potassium-evoked [^3^H]-serotonin release [21]. Here, we tested whether selective deletion of GSK3β from serotonin neurons alters 5-HT1B and 5-HT1A autoreceptor-regulated serotonin release in the cerebral cortical slices. In the absence of 5-HT1B or 5-HT1A receptor agonists, potassium-evoked ^3^H-serotonin release was almost identical in the cortical slices of WT and snGSK3β-KO mice (Figure 5A). In WT mice, anpirtoline caused a significant 33% reduction of potassium-evoked serotonin release. In contrast, anpirtoline only caused a 17% reduction of serotonin release in snGSK3β-KO mice, significantly less than in WT mice (F(2,26)_treatment_ = 13.31, *p*<0.0001; F(1,26)_genotype_ = 5.11, *p* = 0.032, F(2,26)_interaction_ = 1.378, *p* = 0.27 respectively; Figure 5B). The 5-HT1A receptor agonist 8-OH-DPAT also caused a less robust, but significant 22% reduction of serotonin release in WT mice. This small effect was retained in snGSK3β-KO mice where 8-OH-DPAT caused a 20% reduction of serotonin release.

### 5. Regulation of open field activity by 5-HT1 receptors in snGSK3β-KO mice

**Figure 6:**
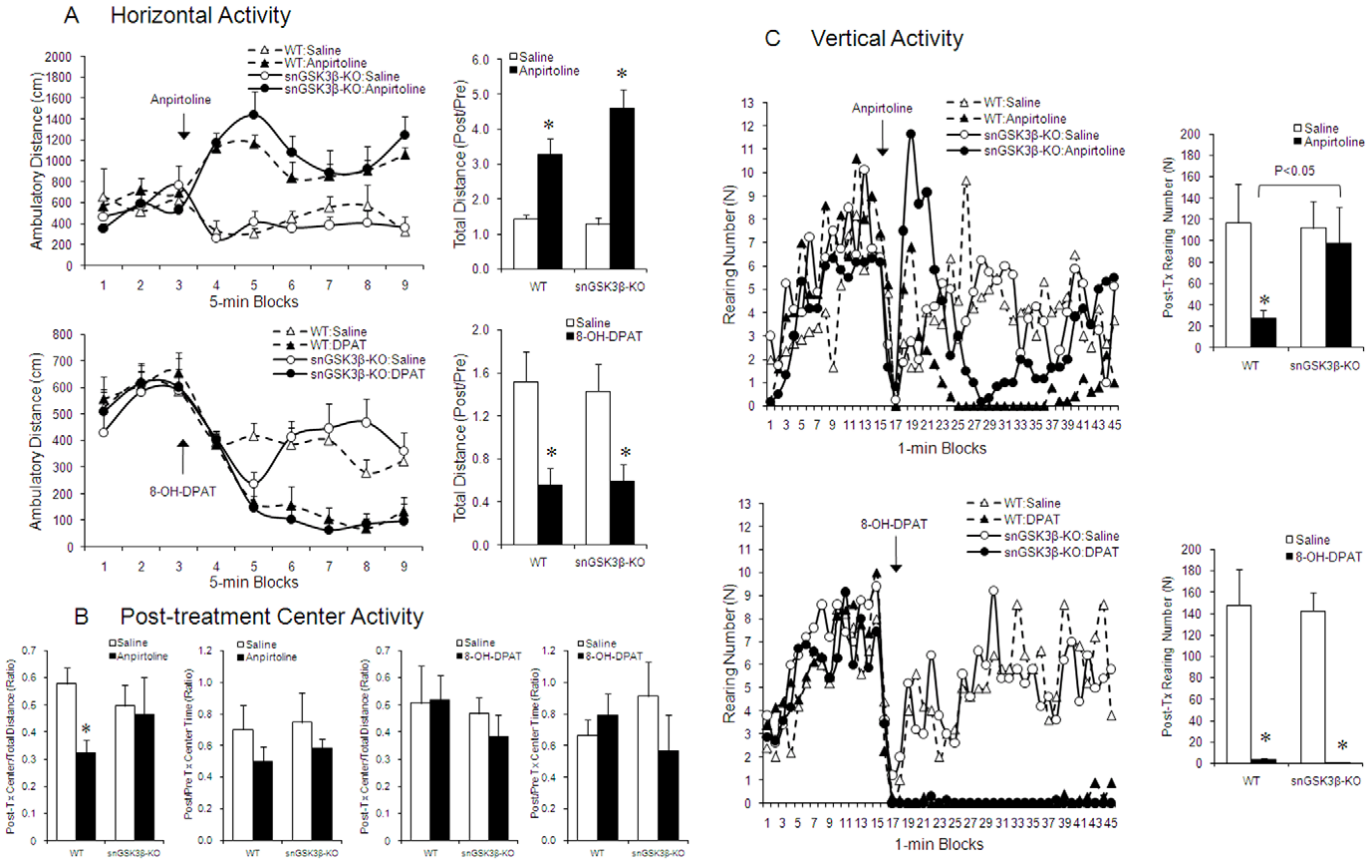
Response of snGSK3β-KO mice to 5-HT1B and 5-HT1A receptor agonists in the open field test. WT and snGSK3β-KO mice were accommodated in the open field plateform for 15 min prior to giving anpirtoline (4 mg/kg, i.p.) or 8-OH-DPAT (1 mg/kg, i.p.) for 30 min. (A) Horizontal activity was demonstrated by an average time course 15 min before till 30 min after drug treatment. Total post-treatment travel distance shown in bar graphs was calculated against pre-treatment baseline activity. (B) Center activity was expressed by distance traveled to the center (left) and center time (right) for each drug treatment. (C) Vertical activity was recorded as numbers of rearing at each min throughout the 45-min test. Total post-treatment vertical activity was shown in the bar graphs. Statistical analysis of each type of activity was conducted using pooled pre- and post-treatment summary data shown in bar graphs. Mean ± SEM, n = 6 in each treatment. *p<0.05 in LSD post hoc of two way ANOVA.

Activation of 5-HT1B receptors is associated with elevated locomotor activity, which is often measured by the horizontal activity in the open field [39], [40]. When this activity was tested in the WT and snGSK3β-KO mice, baseline activity did not differ between the two genotypes. Systemically administered anpirtoline (4 mg/kg, i.p.) induced a significant increase in total horizontal travel distance, and this effect of anpirtoline was similar in WT and snGSK3β-KO mice (two way ANOVA; F(1,16)_treatment_ = 37.256, *p*<0.001; F(1,16)_genotype_ = 1.928, *p* = 0.184; F(1,16)_interaction_ = 3.05, *p* = 0.1; Figure 6A, upper panel). In contrast, 5-HT1A receptor agonist 8-OH-DPAT (1 mg/kg, i.p.) caused a significant reduction in horizontal activity, and this effect of 8-OH-DPAT was also similar in WT and snGSK3β-KO mice (two way ANOVA; F(1,20)_treatment_ = 19.98, *p* = 0.0002; F(1,20)_genotype_ = 0.02, *p* = 0.887; F(1,20)_interaction_ = 0.09, *p* = 0.7696; Figure 6A, lower panel).

When center activity was tested, anpirtoline was found to significantly reduce the travel distance to the center of the open field arena (two-way ANOVA; F(1,18)_treatment_ = 4.56, *p* = 0.0467; F(1,18)_genotype_ = 0.06, *p* = 0.8116; F(1,18)_interaction_ = 1.09, *p* = 0.3137; Figure 6B, first panel from left), but this effect of anpirtoline was not significant in snGSK3β-KO mice (p>0.05; Figure 6B, first panel from left). The genotype effect of anpirtoline on center activity was only notable for travel distance to the center. Although there was a tendency for reduced center time in WT mice by anpirtoline, this effect was not significant in either genotype (two-way ANOVA; F(1,16)_treatment_ = 2.11, *p* = 0.1658; F(1,16)_genotype_ = 0.25, *p* = 0.6226; F(1,16)_interaction_ = 0.02, *p* = 0.8869; Figure 6B, second panel from left). 5-HT1A receptor activation by 8-OH-DPAT did not affect center activity in either WT or snGSK3β-KO mice (two-way ANOVA for center travel distance: F(1,21)_treatment_ = 0.79, *p* = 0.3827; F(1,21)_genotype_ = 0.98, *p* = 0.3339; F(1,21)_interaction_ = 0.14, *p* = 0.7137; Figure 6B, third panel from left; two-way ANOVA for center time: F(1,29)_treatment_ = 0.39, *p* = 0.5397, F(1,29)_genotype_ = 0.00, *p* = 0.9466; F(1,29)_interaction_ = 1.78, *p* = 0.1924; Figure 6B, first panel from right).

Another behavioral component in the open field is vertical activity, which has been used to monitor behaviors related to exploration or anxiety [41]. The time course showed that baseline vertical activity varied at each recorded time point in both genotypes. While there was an observable tendency for anpirtoline to cause a sustained reduction in vertical activity throughout the 30 min treatment (Figure 6C, upper left panel), the effect of anpirtoline in snGSK3β-KO mice appeared to include a robust increase in the initial vertical activity followed by a transient reduction of activity that did not sustain. To summarize the overall effect of anpirtoline in vertical activity, we analyzed the ratio of overall post-treatment to pre-treatment effect. The vertical activity in saline-treated animals was similar between WT and snGSK3β-KO mice. Anpirtoline significantly reduced overall vertical activity during the 30 min post-treatment time. The treatment effect was significantly different in WT mice, whereas was not different in snGSK3β-KO mice (two-way ANOVA; the main effect and interaction: F(1,16)_treatment_ = 6.28, *p* = 0.0234, F(1,16)_genotype_ = 0.03, *p* = 0.8727, F(1,16)_interaction_ = 3.34, *p* = 0.0863; Figure 6C, upper right panel). Similar to anpirtoline, the 5-HT1A receptor agonist 8-OH-DPAT also caused a significant reduction in vertical activity in WT mice. However, this effect of 8-OH-DPAT in snGSK3β-KO mice was almost identical to that found in WT mice (two-way ANOVA; F(1,16)_treatment_ = 89.69, *p*<0.0001; F(1,16)_genotype_ = 0.06, *p* = 0.8098; F(1,16)_interaction_ = 0.02, *p* = 0.8923; Figure 6C, lower right panel). Therefore, vertical activity was similarly suppressed by 5-HT1B and 5-HT1A receptors, but GSK3β in serotonin neurons only influences the effect of 5-HT1B receptor-, not 5-HT1A receptor-regulated vertical activity.

### 6. Effect of anpirtoline on tail suspension test (TST) in snGSK3β-KO mice

**Figure 7:**
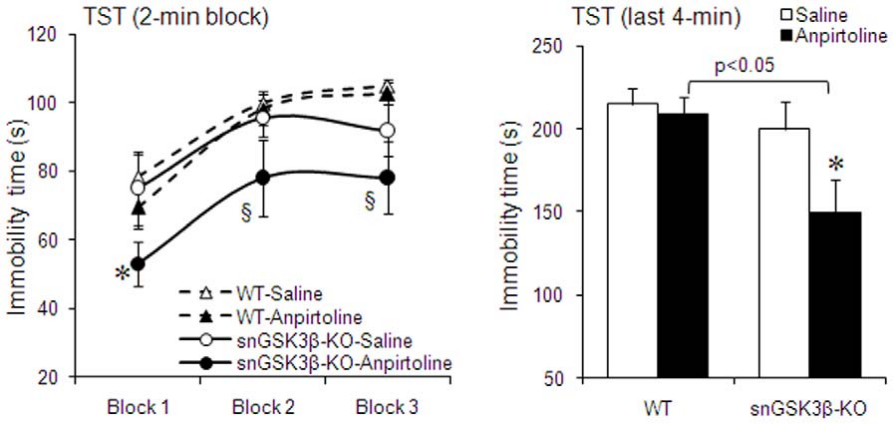
The anti-immobility effect of 5-HT1B receptor agonist in the TST. WT and snGSK3β-KO mice were treated with anpirtoline (4 mg/kg, i.p.) or saline for 30 min before they were subject to the TST. Immobility time was recorded and expressed by each 2 min blocks (left panel) and by the last 4 min of the 6-min test (right panel). n = 8–12 in each treatment group. Immobility time was recorded and expressed by the last 4 min of the 6-min test. Mean ± SEM, n = 5–6 in each treatment group. *p<0.05 in LSD post hoc of two way ANOVA when treatment was compared to baseline in the same genotypes, ^§^ *p*<0.05 in LSD post hoc of two way ANOVA when treatment in snGSK3β-KO mice was compared to the same treatment in WT mice.

5-HT1B receptor agonists can reduce immobility in TST [42], [43], and we previously found that GSK3 inhibitors intensified the anti-immobility effect of anpirtoline in TST [21]. However, intracerebroventricular administered GSK3 inhibitors would inhibit GSK3 in all surrounding neurons with little selectivity for specific neuron types. Here, we tested if deletion of GSK3β in serotonin neurons enhances the anti-immobility effect of anpirtoline in snGSK3β-KO mice. In WT mice, a relatively low dose of anpirtoline (4 mg/kg, i.p.) did not cause a significant reduction in immobility in TST (Figure 7). In snGSK3β-KO mice, the baseline TST immobility time was almost identical to that of WT mice (two-way ANOVA; F(1,50)_genotype_ = 1.92, *p* = 0.1718; F(2,50)_block_ = 8.09, *p* = 0.0009; F(2,50)_interaction_ = 0.37, *p* = 0.6926; Figure 7). However, the same dose of anpirtoline caused 20% reduction of immobility in snGSK3β-KO mice, which was a significant anti-immobility effect comparing to baseline immobility in snGSK3β-KO mice (two-way ANOVA; F(1,47)_treatment_ = 5.69, *p* = 0.0211; F(2,47)_block_ = 3.85, *p* = 0.0281; F(2,47)_interaction_ = 0.10, *p* = 0.9037; Figure 7) as well as to immobility in anpirtoline-treated WT mice (two-way ANOVA for the 2-min block: F(1,60)_genotype_ = 12.22, *p* = 0.0009; F(2,60)_block_ = 10.26, *p* = 0.0001; F(2,60)_interaction_ = 0.16, *p* = 0.8536; two-way ANOVA for the last 4-min block: F(1,36)_treatment_ = 4.23, *p* = 0.0464; F(1,36)_genotype_ = 5.68, *p* = 0.0226; F(1,36)_interaction_ = 1.77, *p* = 0.1918; Figure 7).

## Discussion

The results of this study demonstrate that GSK3β modulates serotonin neuron function. This study showed that one of its effects in serotonin neurons is to selectively modulate activity of 5-HT1B autoreceptors that impact 5-HT1B receptor-regulated serotonin neuron firing, cAMP production, serotonin release, and behaviors. Observation of this study, however, does not exclude that the multi-functional GSK3β may also affect other functions of serotonin neurons, which remain to be investigated.

The serotonin neuron-selective GSK3β knockout is the first model to specifically delete GSK3β in a single type of neurons in the central serotonin system. It provides a tool to sort out the effect of GSK3β on type 1 serotonin autoreceptors that attribute to the complex physiological and behavioral actions of serotonin. In this study, we focused on the beta-isoform of GSK3 (GSK3β) because we previously established that GSK3β is a direct modulator of 5-HT1B receptors, whereas GSK3α has little effect on 5-HT1B receptors [21], [22]. In earlier studies, complete inactivation of GSK3β was found to cause late embryonic lethality [34], but GSK3β knockout by tissue-selective recombination of floxed GSK3β, such as in the liver [32] and the renal collecting duct [44], does not profoundly affect survival. We therefore utilized GSK3β-floxed animals to develop snGSK3β-KO mice. We elected to use ePet1-directed Cre recombinase [33] to delete GSK3β in serotonin neurons because Pet1 ETS gene expression is restricted in the entire rostrocaudal extent of serotonergic hindbrain raphe nuclei, and Pet1 RNA colocalizes with TpH2-positive neurons but not with non-serotonergic cells in raphe nuclei [45], [46]. The reduction of GSK3β expression in TpH2-expressing serotonin-synthesizing neurons was significant when tested by immunohistochemistry, but GSK3β was not completely deleted from the midbrain raphe. The residual GSK3β in raphe could be due to expression in Pet-1 non-expressing serotonin neurons or in non-serotonin cells, or due to an incomplete knockout of GSK3β in serotonin neurons, which remains to be determined. GSK3β expression in the cerebral cortex was not significantly changed in snGSK3β-KO mice, which is likely due to the scattered axon distribution of small numbers of serotonin neurons in brain areas outside the raphe nucleus. We also did not detect a difference of the expression of 5-HT1B and 5-HT1A receptors in the midbrain and cerebral cortex (data not shown). Although we could not rule out technical limitations of separating serotonin autoreceptors from heteroreceptors in these brain regions, in our previous reports [21], [22], GSK3 inhibitors or knock-down of GSK3β significantly altered the activity of 5-HT1B receptors when the level of exogenously expressed 5-HT1B receptors remains the same. The similarities in serotonin neuron numbers, brain serotonin content, potassium-evoked serotonin release, animal survival, physiological conditions, and basic movement between WT and snGSK3β-KO mice suggest that embryonic deletion of GSK3β does not grossly affect the presence of serotonin neurons, serotonin synthesis capacity, and some basic functions of serotonin. Since Pet-1 expresses in serotonin neurons at embryonic day-12.5, one day prior to the onset of serotonin synthesis in these cells [45], knockout GSK3β at this stage of development may not significantly affect formation of serotonin neuron. This would be different from systemic GSK3β knockout that has no GSK3β expression at the beginning of neuron development [34]. Alternatively, the remaining GSK3α in these neurons may compensate for the basal activity of GSK3 in serotonin neurons. The new snGSK3β-KO mouse model is thus suitable to address the central question of this study – the impact of GSK3β on 5-HT1 autoreceptor activity.

In this study, electrophysiological recording of serotonin neuron firing revealed a specific modulating effect of GSK3β. The basal serotonin neuronal firing rate is higher in snGSK3β-KO mice in both physiological and high potassium conditions, which is likely the first evidence showing that active GSK3β contributes to the threshold of serotonergic neuronal activity under physiological conditions. It also raises the possibility that during pathological conditions with GSK3β hyperactivity, which may occur when the inhibitory control of GSK3β is impaired in neuropsychiatric disorders [47], appropriate therapeutic inhibition of GSK3β may restore serotonergic neuronal activity. The complete suppression of spontaneous serotonin neuron firing by serotonin and 5-HT1A receptor activation [48] was not altered by deleting GSK3β, which is in agreement with our molecular studies showing that activation of 5-HT1A receptors is not GSK3β-dependent [21], [22]. The underlying mechanism for the inability of 5-HT1B receptor activation to alter the basal spontaneous serotonin neuron firing is not clear, but we speculate that the axon terminal 5-HT1B autoreceptors are likely involved in regulating active serotonin neuron firing. High potassium was applied to depolarize neurons [37], and as shown it increased serotonin neuron firing, which then allowed measurement of 5-HT1B autoreceptor-induced negative regulation of neuron firing and to test whether GSK3β influenced the effect of active 5-HT1B autoreceptors. Since potassium-induced serotonin neuron firing was higher in snGSK3β-KO mice, we calculated the effect of anpirtoline as a percentage of the baseline firing rate. Indeed, a diminished effect of anpirtoline in snGSK3β-KO mice suggests that the effect of 5-HT1B autoreceptors on serotonin neuron activity in the high potassium condition is modulated by GSK3β.

In our previous studies, 5-HT1B receptor-induced inhibition of cAMP production in cerebral cortical slices was abolished by treatment with GSK3 inhibitors [21], [22]. Somewhat unexpectedly, the effect of a low concentration of anpirtoline on cAMP was not altered in snGSK3β-KO mice, whereas the effects of higher concentrations of anpirtoline were reduced. Our antagonist experiment confirmed that the higher concentrations of anpirtoline used in the study were selective to 5-HT1B receptors, and thus, this is likely an effect of GSK3β on 5-HT1B autoreceptors. However, since cAMP measured in cortical slices includes cAMP produced by all types of cells, and there are 5-HT1B autoreceptors and heteroreceptors in the cerebral cortex, this is not a serotonin neuron-specific measurement. Therefore, the lack of effect seen at lower concentration of anpirtoline may be due to activation of 5-HT1B heteroreceptors located in other types of cells that retain normal expression of GSK3β. Alternatively, a subtype of high affinity 5-HT1B autoreceptors may exist in serotonin neurons, the activity of which is independent of GSK3β. Nevertheless, the significantly reduced response to higher concentrations of anpirtoline in snGSK3β-KO mice is in support of a modulating effect of GSK3β on 5-HT1B autoreceptors in serotonin neurons. Likewise, the unaltered response of cAMP to the 5-HT1A receptor agonist 8-OH-DPAT in snGSK3β-KO mice provides additional evidence that GSK3β selectively modulates the activity of 5-HT1B autoreceptors and not 5-HT1A autoreceptors.

Regulation of serotonin release is a specific function of serotonin neurons, and we have previously found that GSK3 inhibitors were able to abolish anpirtoline-induced inhibition of potassium-evoked serotonin release in cortical slices [21]. The reduced effect of anpirtoline on serotonin release in snGSK3β-KO mice was less robust than GSK3 inhibitors, which may be explained by incomplete knockout of GSK3β in some serotonin neurons as shown in Figure 1, but this effect on 5-HT1B autoreceptors was substantially different from that on 5-HT1A autoreceptors as 8-OH-DPAT-induced inhibition of serotonin release in cortical slices was not altered in snGSK3β-KO mice. Since activation of 5-HT1B autoreceptors causes negative feedback inhibition of serotonin release, deleting GSK3β from serotonin neurons could potentially reduce the activity of 5-HT1B autoreceptors and thus maintain sufficient serotonin supply in synapses to regulate functions of other neurons.

With the mechanistic model presented in this study, it appears that GSK3β has little interaction with 5-HT1A receptors. It should be clarified that 5-HT1A receptors can regulate GSK3β activity in mouse brain, wherein activation of 5-HT1A receptors results in phosphorylation of GSK3β at its serine-9 residue [5], [49] and therefore, inactivation of GSK3β [23]. The potential impact of GSK3β in mediating 5-HT1A autoreceptor signaling in serotonin neurons has not been studied, but the snGSK3β-KO mouse model is not designed to study this role of GSK3β because GSK3β is abolished in these mice, thus GSK3β-dependent 5-HT1A autoreceptor actions may have been maximized, instead of diminished [8].

In animal models, 5-HT1B receptors have effects in regulating behaviors relevant to mood, anxiety, hyperactivity, aggression, reward, perseveration, and cognition [26], [50], [51], [52], [53]. However, the behavioral effects of 5-HT1B receptors are diversely mediated by their autoreceptors and heteroreceptors [26], which appears to be a major challenge in understanding the behavioral effects of brain 5-HT1B receptors. Since the snGSK3β-KO mouse model only deletes GSK3β in serotonin neurons, it allows testing of the behavioral effects of 5-HT1B autoreceptors.

5-HT1B receptor agonists are known to increase horizontal activity in the open field test [39], [40]. However, this well-known effect of 5-HT1B receptors is less valuable in testing the effect of GSK3β on 5-HT1B receptors because our previous study showed that GSK3β inhibitors did not affect this behavioral effect of 5-HT1B receptors [21]. Indeed, GSK3β knockout from serotonin neurons had no effect on anpirtoline-induced horizontal activity. Although not fully understood, previous studies suggest that this particular behavior is mostly regulated by 5-HT1B heteroreceptors [39], [54] and is affected by β-arrestins associated with 5-HT1B receptors [21]. In contrast, activation of 5-HT1A receptors by 8-OH-DPAT reduced horizontal activity. This finding is the same as reported by others [55], supporting the validity of our test. Since this effect of 8-OH-DPAT was not altered in snGSK3β-KO mice, it does not distinguish between non-effectiveness of GSK3β on 5-HT1A autoreceptors or whether this is a function of 5-HT1A heteroreceptors. In addition, the opposite effects of 5-HT1B and 5-HT1A receptors on horizontal activity do not allow testing of the differential impact of GSK3β on a similar effect of the two subtypes of 5-HT1 receptors.

When center travel distance was calculated as the ratio of total horizontal travel distance, anpirtoline reduced center activity. This effect of anpirtoline likely involves activation of 5-HT1B autoreceptors since it was completely abolished in snGSK3β-KO mice. However, this appears to be a small effect since anpirtoline appeared to increase the total travel distance if the ratio was not analyzed. In addition, time in the center was also less affected by anpirtoline, which was possibly due to an overall increase in total horizontal activity. Additionally, this behavior could not compare the effect of GSK3β on 5-HT1B and 5-HT1A receptors, because 8-OH-DPAT was without an effect on center activity.

Interestingly, both anpirtoline and 8-OH-DPAT significantly reduced vertical activity in the open field test. This effect may represent an acute effect of these two 5-HT1 receptor subtypes in reducing exploration or provoking anxious behaviors [41]. Since the effect of anpirtoline was drastically altered in snGSK3β-KO mice, the result suggests that this behavioral effect of 5-HT1B receptors involves activation of its autoreceptors. In the absence of GSK3β in serotonin neurons, 5-HT1B autoreceptors are less functional [21], [22], resulting in excessive exposure of sensitive neurons to unchecked serotonin signals. The time course of vertical movement recorded at each min of a 30-min post-treatment time revealed that snGSK3β-KO mice had a very different response pattern to anpirtoline, from an initial over-reactivity to a transient suppression, but the response to anpirtoline was not sustained as seen in WT mice. This behavioral result builds on our previous impression that GSK3β does not function as an endogenous, full activator of 5-HT1B receptors; rather, GSK3β is a critical modulator for fine-tuning the activity of 5-HT1B receptors [8]. In striking contrast, the effect of 8-OH-DPAT on vertical activity was the same in WT and in snGSK3β-KO mice, again suggesting that GSK3β does not directly affect 5-HT1A receptor activity.

5-HT1B receptor agonists can reduce immobility in TST and forced swim test [42], [43]. This behavioral effect per se is thought to be a function of 5-HT1B heteroreceptors located within non-serotonin neurons [42], but the effect usually requires high dosage of 5-HT1B receptor agonist when administered via a systemic route, presumably because simultaneous activation of 5-HT1B autoreceptors suppresses serotonin release and dampens the heteroreceptor-mediated behavioral effect. In reverse, enhancing serotonin release by ablating 5-HT1B autoreceptor action reportedly facilitates 5-HT1B receptor agonist-induced reduction of immobility [42], [43]. Utilizing the snGSK3β-KO mouse model that is proposed to affect only the function of 5-HT1B autoreceptors, we showed that disrupting the sensitivity of 5-HT1B autoreceptors to its agonist facilitates its anti-immobility effect. Indeed, the result obtained in snGSKβ-KO mice was similar to our previous finding using GSK3 inhibitors, but better demonstrates an impact of GSK3β on 5-HT1B autoreceptors. We hypothesize that when 5-HT1B autoreceptor activity is suppressed in snGSK3β-KO mice, not only does anpirtoline mainly activates 5-HT1B heteroreceptors, but serotonin release is also enhanced in the absence of feedback inhibition by 5-HT1B autoreceptors. Consequently, serotonin neurotransmission is enhanced, which would then facilitate the anti-immobility effect of anpirtoline.

Overall, knockout of GSK3β in serotonin neurons provides a means to examine the activity and function of 5-HT1B autoreceptors without interfering with the activity of 5-HT1B heteroreceptors, which has been a challenge using conventional pharmacological approaches. Findings of this study provide new evidence that GSK3β modulates 5-HT1B autoreceptor-mediated signaling, physiology, and behavior, which entitles GSK3β as an active modulator of serotonin neurotransmission. This may partly explain the diverse effects of GSK3β in behaviors related to emotion, anxiety, and activity [47]. Noticeably, among reported *in vivo* effects of lithium [56], [57], [58], [59], several earlier studies showed that lithium inhibits 5-HT1B receptors, and it was proposed that 5-HT1B receptors may be a therapeutic target of lithium [60]. With lithium being a GSK3 inhibitor [9] and our previously finding that lithium disrupts the direct interaction between GSK3β and 5-HT1B receptors in transfected cells [22], it is probable that the effect of lithium on 5-HT1B receptor function is the result of its inhibition of GSK3β. Since lithium is a therapeutic agent in neuropsychiatric disorders [61], finding of this study warrants future studies to investigate therapeutic approaches that disrupt the interaction between GSK3β and 5-HT1B autoreceptors to facilitate serotonin neurotransmission for the treatment of neuropsychiatric disorders.

## Materials and Methods

### Chemicals

Anpirtoline, 1′-Methyl-5-[[2′-methyl-4′-(5-methyl-1,2,4-oxadiazol-3-yl)biphenyl-4-yl]carbonyl]-2,3,6,7-tetrahydrospiro[furo[2,3-f]indole-3,4′-piperidine (SB224289), (Tocris, Ellisville, MO); serotonin, 8-hydroxy-*N*,*N*-dipropyl-2-aminotetralin (8-OH-DPAT), N-[2-[4-(2-Methoxyphenyl)-1-piperazinyl]ethyl]-N-2-pyridinylcyclohexanecarboxamide (WAY100635), (Sigma-Aldrich, St. Louis, MO); [^3^H]-serotonin ([^3^H]-5-hydroxytryptamine trifluoroacetate, specific activity: 100 Ci/mmol; stock concentration: 1 mCi/ml; PerkinElmer, Waltham, MA). Chemicals and drugs are dissolved in distilled water or saline before they were used for experiments.

### Animals

The Institutional Animal Care and Use Committee at the University of Alabama at Birmingham approved animal use in this study.

Homozygous GSK3β-flox/flox mice in a mixed C57BL/6 and 129 background [32] were cross-bred with ePet1-Cre mice on a C57BL/6 background [33] to generate homozygous GSK3β-flox/flox:ePet1-Cre (snGSK3β-KO) and littermate wild type (WT, GSK3β-flox/flox) mice. Genotypes were confirmed by Polymerase Chain Reaction (PCR) of tail DNA using primers 5′-GGGGCAACCTTAATTTCATT and 5′-TCTGGGCTATAGCTATCTAGTAACG for GSK3β, and 5′-AAAATTTGCCTGCATTACCG-3′ and 5′-ATTCTCCCACCGTCAGTACG-3′ for Cre (Figure 1A). TpH2 R439H knockin mice were described previously [11].

Body Weight and temperature [62] were recorded from 4–12 weeks of age once per week between 15:00 and 17:00. Scout™Pro (SP 601) scale (Chaus Corporation, Pine Brook, NJ USA) was used to measure body weight. Lubricated Thermalert TH-5 probe (Physitemp Instrument Inc., Clifton, NJ 07013) was inserted in the rectum for 15–20 sec to obtain stable recording of body temperature. Defecation [62] was recorded at 12 weeks of age by counting numbers of feces produced by each mouse during a 24-hour period.

Experimental mice were housed 4–5 per cage with free access to food and water in a 12-hour light/dark cycle animal facility. Adult (8–14 weeks old) male homozygous snGSK3β-KO mice and WT (GSK3β-flox/flox) mice were used for physiology, bioassays, and behavior tests. Since these mice were derived from GSK3β-flox/flox breeders with a mixed strain background, to minimize the heterogeneous responses, each experiment was conducted using littermate snGSK3β-KO mice and WT mice.

### Immunohistochemistry of TpH2 and GSK3β

Mice were anesthetized with ketamine and xylazine (100 mg/kg∶10 mg/kg) for transcardiac perfusion with 4% paraformaldehyde (PFA)/0.1M PBS (pH 7.4). Perfusion-fixed brains were immersed overnight in 30% sucrose, imbedded in the Tissue-Tek Optimal Cutting Temperature (O.C.T.) compound (Sakura Finetek, Torrance, CA), and frozen. Coronal and sagital brain sections (20 µm) were prepared on the Leica CM1850 cryostat slicer (Leica Microsystem, Buffalo Grove, IL). The coronal midbrain sections corresponding to Bregma: −3.6 mm to −5.2 mm were labeled with antibodies to GSK3β (BD Biosciences, Franklin Lakes, NJ) and to tryptophan hydroxylase (TpH2) (Novus Biologicals, Littleton, CO), and the sagital sections including the forebrain were labeled with anti-GSK3β. Sections were then reacted with the horseradish peroxidase-conjugated anti-mouse or anti-rabbit serum, treated with the Tyramide Signal Amplification (TSA) kit (Perkin Elmer, Waltham, MA), and counter-stained with the nuclear marker Hoechst 33,258 (Sigma-Aldrich, St. Louis, MS). Immunofluorescence of brain sections was viewed and captured with the Leica DM6000B digital confocal microscope (Leica Microsystem, Buffalo Grove, IL). The number of TpH2-positive cells and GSK3β/TpH2 co-stained cells were counted manually by an unbiased observer blind to genotypes. Photographed fields (63x magnification) from each of the ventral, dorsal, and dorsal-lateral raphe nucleus were counted. Three animals from each genotype with 1–2 brain slices from each animal were used for cell counting. Only cells with a nucleus (Hoechst 33,258 stained) are included for cell counting. GSK3 expression in serotonin neurons were expressed by co-stained cells as percent of total TpH2-positive cells in each area. Cell counts were confirmed by counting each field twice from the left to the right.

### Immunohistochemistry of brain serotonin

All mice used in this experiment were 3 months old females, including TpH2 R439H knockin homozygote and littermate WT [11], and snGSK3â-KO and littermate WT mice. After perfusion fixation as described above, sagittal sections (50 µm) of the frozen brain were made by vibratom the day of staining. The selected slices were incubated in PBS, 3% BSA and 0,5%Triton100X, overnight at 4°C in the presence of rabbit anti-serotonin antibody (1∶100, S5545 Sigma-Aldrich). After washing in PBS (3 times, 10 min), the sections were incubated for 2 hours at room temperature with anti-rabbit IR800CW from (1∶1000, Licor, Lincoln, NE). They were washed and mounted on slides with Prolong Gold. Tissue sections were imaged at a 21 µm resolution with the Odyssey Licor imager [35]. Comparative quantification was always made on the same scan and regions of interest (ROI) used for quantification of the same area for the same brain region from section to section. Six tissue sections from two animals of each genotype (3 sections per animal) were used for analysis.

### Raphe serotonin neuron firing

For electrophysiology experiments, WT and snGSK3β-KO mice were euthanized by decapitation and brains were removed and blocked in cold sucrose-substituted saline solution [36], [63]. Briefly, coronal midbrain slices (200 µm) containing the dorsal raphe nucleus were sliced on a Vibroslicer at 4–10°C and maintained in 50% sucrose saline/50% normal saline solution for 10 min at room temperature followed by 100% normal saline solution maintained at 34±0.5°C. Neurons were visualized with an Axio Examiner microscope (Carl Zeiss Inc., Thornwood, NY) equipped for near-IR digital image correlation. Extracellular patch electrodes with a pipette resistance of 3 to 4 MΩ were filled with filtered, normal Hepes solution. Firing rate frequency was measured as the average of a 30-sec recording. All neurons were treated first with serotonin (40 µM) for 2 min, followed by at least 10 min washout to allow for complete firing rate recovery, and then treated with anpirtoline (10 µM) or 8-OH-DPAT (1 µM) for 2 min. For high potassium condition [37], neurons were given potassium chloride (KCl, 10 mM) for 2 min, followed by a 2 min application of anpirtoline (5 µM). All solutions contained 3 µM phenylephrine hydrochloride and 24 µM L-tryptophan to maintain the spontaneous firing rate in the absence of noradrenergic tone and tryptophan availability [64].

### Cyclic AMP (cAMP) assay

Cerebral cortical slices from WT and snGSK3β-KO mice were prepared as previously described [22], followed by incubation with pharmacological agents. Cortical slices were treated with or without 5-HT1B receptor antagonist SB224289 or 5-HT1A receptor antagonist WAY100635 for 15 min before treatment with 5-HT1B receptor agonist anpirtoline or 5-HT1A receptor agonist 8-OH-DPAT for 30 min, followed by forskolin (10 µM) treatment for 15 min. After pharmacological treatments, cortical slices were lysed in a buffer containing 2.5% dodecyltrimethylammonium bromide, and the level of cAMP in the lysate was measured using an enzyme immunoassay kit (Direct BioTrak, Amersham/GE). Cyclic AMP production was calculated against a cAMP standard, and normalized by total protein content in each sample (pmol/mg) [21], [22].

### Serotonin release

Cerebral cortical slices from WT and snGSK3β-KO mice were prepared as previously described [21]. Cortical slices were labeled with [^3^H]-serotonin (1.3 µCi/ml) in Krebs buffer [121 mM NaCl, 1.2 mM MgCl_2_, 1.3 mM CaCl_2_, 25 mM NaHCO_3_, 1.0 mM NaH_2_PO_4_, 10 mM glucose, 13.5 mM KCl, 50 µM l-tryptophan, 50 µM l-tyrosine, 10 µM nomifensine malate (dopamine reuptake inhibitor), and 10 µM imipramine (serotonin reuptake inhibitor)] with 95% O_2_ and 5% CO_2_ at 37°C for 30 min, transferred into the perfusion chambers (10∼11 slices/chamber) and superfused with aerated (95% O_2_ and 5% CO_2_) and preheated (37°C) Krebs buffer. The flow rate was kept at 0.5 ml per minute by a BIO-RAD multichannel peristaltic pump (Econo Pump, USA). Beginning at 61 min of perfusion, the superfusion fluid was collected at 0.5 ml/min. Brain slices were stimulated with potassium chloride (KCl, 50 mM) at 63 min and at 83 min, respectively. For anpirtoline or 8-OH-DPAT treatment, the perfusion buffer was replaced at 78 min by fresh Krebs buffer containing anpirtoline or 8-OH-DPAT (5 µM). Collection of superfusion fluid continued until 98 min. At the end of the experiment, the brain slices from each chamber were collected and homogenized in 200 µl of 1N HCl containing 0.1% Triton X-100. Superfusion fluid from each fraction and tissue homogenate (200 µl) was mixed with 3 ml scintillation liquid (Universol, MP), and radioactivity was counted by a scintillation spectrometry (Multi-Purpose Scintillation Counter, LS-6500, Beckman). To calculate [^3^H]-serotonin release, the radioactivity from fractions collected at 61–63 min were averaged as a baseline for the first KCl stimulation, and fractions at 81–83 min were averaged for the second KCl stimulation. The radioactivity of all fractions was calculated as: CPM x 100/baseline CPM. Data were processed by OringPro 8.1, and potassium-evoked [^3^H]-serotonin release was represented by the peak area surrounded by the fitting line and baseline. Effect of a drug was determined by the ratio of the second overflow (P2) to the first overflow (P1) [21].

### Rotarod Test

Rotarod was used to measure motor coordination [65]. Mice were trained on the Acceler Rota-Rod (Ugo Basile, Collegeville, PA) with a fixed speed (10 rpm) for 10 min each time, 3 times per day for 3 continuous days. Fourteen days later the same mice were trained again on the rotarod with an accelerating speed 3 times in one day. The motor ability was tested on the next day with the same accelerating speed. Latency to drop from the rotarod was recorded as the index of motor ability.

### Open field test

The locomotor activity was tested in a plexiglas open field (Med Associates, St. Albans, VT), and activity was monitored using the activity monitoring software (Med Associates, St. Albans VT) [21], [66]. Mice were allowed to habituate in the open field for 15 min before drug treatment, followed by an additional 30 min testing in the open field. Activity was measured by recording horizontal activity, center activity, and vertical activity. The post-treatment horizontal activity was calculated as the ratio of post-treatment over pre-treatment horizontal travel distance. The post-treatment center activity was calculated as the distance traveled to the center over total horizontal travel distance. The vertical activity was recorded by the total numbers of rearing.

### Tail suspension test (TST)

TST was conducted using an automated testing system (Med Associates Inc, St. Albans, VT) [21], [66]. Movement is measured for 6 min, and the immobility time was recorded with the Med Associates software and calculated as the time the force of movement was below a preset threshold. Immobility was recorded as each 2-min bin and the last 4 min of testing. For drug treatment, anpirtoline or saline was given for 30 min before testing.

### Data Analysis and Statistics

All experiments and treatments were repeated for statistical analysis. Data were analyzed with Student's t-test, one-way or two-way analysis of variance (ANOVA) with LSD post hoc analysis for multiple comparisons, two-way repeated measures ANOVA with Sidak post hoc analysis for multiple comparisons, or mixed models where appropriate. For repeated measures, if sphericity was violated (indicated by a significant Mauchly's W), a Greenhouse-Geisser adjustment of the degrees of freedom was employed. Values are expressed as mean ± SEM and are considered significant when *p*<0.05. SPSS 13.0.0.0, PASW 18.0, and SAS 9.2 software were used for statistical analysis.
